# Mesenchymal stem cells are attracted to latent HIV-1-infected cells and enable virus reactivation via a non-canonical PI3K-NFκB signaling pathway

**DOI:** 10.1038/s41598-018-32657-y

**Published:** 2018-10-02

**Authors:** Partha K. Chandra, Samantha L. Gerlach, Chengxiang Wu, Namrata Khurana, Lauren T. Swientoniewski, Asim B. Abdel-Mageed, Jian Li, Stephen E. Braun, Debasis Mondal

**Affiliations:** 1 Department of Pharmacology, Tulane University School of Medicine, New Orleans, LA USA; 2 Tulane National Primate Research Center, Covington, LA USA; 3 Department of Urology, Tulane University School of Medicine, New Orleans, LA USA; 4 Tulane School of Public Health and Tropical Medicine, New Orleans, LA USA

## Abstract

Persistence of latent HIV-1 in macrophages (MACs) and T-helper lymphocytes (THLs) remain a major therapeutic challenge. Currently available latency reversing agents (LRAs) are not very effective *in vivo*. Therefore, understanding of physiologic mechanisms that dictate HIV-1 latency/reactivation in reservoirs is clearly needed. Mesenchymal stromal/stem cells (MSCs) regulate the function of immune cells; however, their role in regulating virus production from latently-infected MACs & THLs is not known. We documented that exposure to MSCs or their conditioned media (MSC-CM) rapidly increased HIV-1 p24 production from the latently-infected U1 (MAC) & ACH2 (THL) cell lines. Exposure to MSCs also increased HIV-1 long terminal repeat (LTR) directed gene expression in the MAC and THL reporter lines, U937-VRX and J-Lat (9.2), respectively. MSCs exposed to CM from U1 cells (U1-CM) showed enhanced migratory ability towards latently-infected cells and retained their latency-reactivation potential. Molecular studies showed that MSC-mediated latency-reactivation was dependent upon both the phosphatidyl inositol-3-kinase (PI3K) and nuclear factor-κB (NFκB) signaling pathways. The pre-clinically tested inhibitors of PI3K (PX-866) and NFκB (CDDO-Me) suppressed MSC-mediated HIV-1 reactivation. Furthermore, coexposure to MSC-CM enhanced the latency-reactivation efficacy of the approved LRAs, vorinostat and panobinostat. Our findings on MSC-mediated latency-reactivation may provide novel strategies against persistent HIV-1 reservoirs.

## Introduction

The epidemic of acquired immune deficiency syndrome (AIDS) has become an adversary of alarming proportions, with more than 39 million deaths worldwide^1,2^. Approximately 37 million individuals are currently living with human immunodeficiency virus type-1 (HIV-1) and more than two million people are newly infected annually^2^. Although the advent of highly active antiretroviral therapy (HAART) has been very effective in suppressing plasma viral load (PVL)^3^, HIV-1 persistence in latent tissue reservoirs remains a significant challenge to long-term cure^4,5^. In these sequestered anatomical reservoirs, low chronic level of virus production and continuous release of viral factors can occur, which facilitate both disease progression and therapeutic resistance. Therefore, substantial efforts are underway to either utilize a ‘shock’ approach towards continuous reactivation and depletion of the latently-infected cells or to target the host cell signaling pathways that enable an effective ‘lock’ of virus reactivation from reservoirs, and thus, enhance the efficacy of HAART^6–8^. Indeed, several latency reversing agents (LRAs) are being tested clinically that either activate NFκB through protein kinase-C (PKC) signaling pathway or enable epigenetic activation of the HIV-1 provirus using the histone deacetylase inhibitors (HDACi)^6,7^. However, the PKC-inducing LRAs, e.g. prostratin, have shown significant side-effects and the HDACi LRAs, e.g. panobinostat, have not been very effective *in vivo*^8^. Therefore, a better understanding of host factors and/or physiologic signaling pathways that regulate latency and/or reactivation within tissue HIV-1 reservoirs may facilitate the discovery of safer and more effective therapeutic strategies in HIV-positive patients^9,10^.

HIV-1 preferentially infects cells of the lymphoid and myeloid lineages, e.g. T-helper lymphocytes (THLs) and monocytes-derived macrophages (MACs), where the virus integrates into host cell chromosome to form the provirus^11^. Transcriptional activation of this latent provirus is primarily regulated by both viral proteins (e.g. Tat and Nef) and host-cell transcription factors (e.g. NFκB and NFAT) that recognize specific sequences within the HIV-1 long terminal repeat (LTR). Interestingly however, molecular studies on proviral latency and/or reactivation have mostly focused on the latently-infected THLs (memory phenotype)^4,12,13^ despite the fact that numerous studies demonstrate a crucial role of latently-infected MACs^14,15^. Indeed, MACs constitute the earliest targets for infection by the CCR5-tropic HIV-1^16^. Furthermore, due to their ability to transmigrate into tissue compartments, MACs are implicated in viral dissemination to multiple anatomical sites^17,18^. The HIV-1-infected MACs play a predominant role in regulating viral load in gut-associated lymphoid tissues (GALT)^19^. Additionally, in the central nervous system (CNS), perivascular MACs (a.k.a. microglial cells) are a major HIV-1 producing cell^20,21^. Both cell-to-cell and/or cell-to-stroma interactions can regulate immune activation^22,23^ and immunoregulatory properties of mesenchymal stem/stromal cells (MSCs) are now well accepted^24–26^. Indeed, exposure to MSC-secretome was shown to alter the M1 or M2 polarization of macrophages^25^. Since MSCs are present in all known HIV-1 reservoirs, such as lymph nodes, bone marrow, brain, gut and adipose tissue^27,28^, the role of MSCs in regulating viral latency and/or reactivation would be of crucial significance.

Our *in vitro* investigations demonstrated that exposure to conditioned medium (CM) from MSCs (MSC-CM) caused a rapid increase in virus production from two latently infected cell line models, i.e. U1 (monocytic) and ACH2 (T-cell). MSCs showed the ability to migrate towards both U1 and ACH2 cells, and MSC with enhanced migratory ability also retained their latency-reactivation potential. Molecular mechanistic studies clearly showed that virus reactivation by MSC-secreted factors occurred due to the activation of HIV-1 LTR directed gene expression. We documented that MSC-secreted factors activate HIV-1 via a non-canonical signal transduction pathway, which was PI3K- and NFκB-dependent, but AKT-independent. Several clinically tested inhibitors of PI3K (e.g. PX-866) and NFκB, (e.g. CDDO-me) were able to suppress this MSC-mediated latency-reactivation. Most importantly, costimulation with MSC-CM significantly enhanced the latency-reactivation ability of the clinically approved LRA, panobinostat. Our findings on MSC-mediated reactivation of latent HIV-1 may provide novel strategies to eliminate the persistent viral reservoirs in patients.

## Results

### Exposure to ASC-secreted factors enabled HIV-1 reactivation from U1 cells

**Figure 1:**
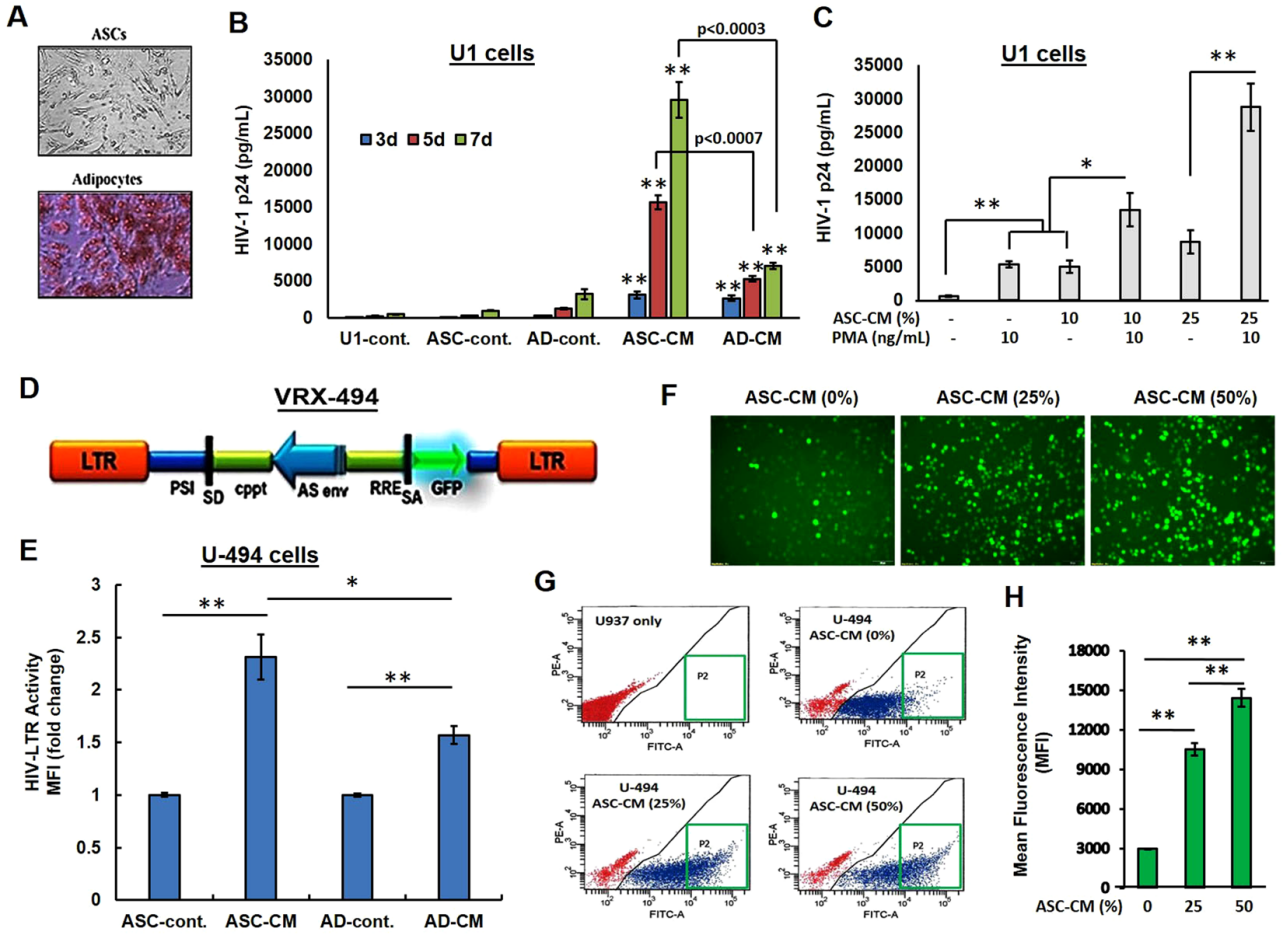
Effect of factors secreted by ASCs and adipocytes on HIV-1 p24 production by U1 cells and HIV-1 LTR function in U-494 cells. (**A**) Representative images of unstained ASCs (top) and oil red-O stained adipocytes (bottom). Adipocyte differentiation was clearly evident. (**B**) ELISA data on HIV-1 p24 production (pg/mL) by U1 cells exposed to conditioned media (CM) from either ASCs (ASC-CM) or adipocytes (AD-CM). Both U1 growth media (U1-cont.), ASC growth media (ASC-cont.) and adipocyte differentiation media (AD-cont.) were used as controls. ASC-CM enabled a more rapid and potent latency-reactivation compared to AD-CM. (**C**) Comparative analysis of HIV-1 p24 levels following exposure of U1 cells to either ASC-CM (10% and 25%) or PMA (10 ng/mL). The ASC secreted factors were as potent as PMA in latency reactivation. (**D**) A schematic of the VRX494 lentivirus (LV) which expresses green fluorescent protein (GFP) under the transcriptional control of HIV-1 long terminal repeat (LTR). In **(E–H)**, the U-494 cells, which were U937 cells stably transduced with LV VRX494, were used to measure HIV-1 LTR directed GFP expression. **(E)** Mean fluorescence intensities (Mean FITC-A) of GFP expression by U-494 cells exposed to either ASC-CM or AD-CM are shown. (**F**) Representative photomicrograph of GFP positive U-494 cells, both unstimulated and following exposure to ASC-CM (25% or 50%). (**G**) Representative flow cytometry panels of increased mean fluorescence intensity (MFI) of the GFP-positive (P2 area) U937 cells (as control) and in both unstimulated and ASC-CM (25% or 50%) stimulated U-494 cells. (**H**) MFIs (n = 3) of GFP expression by U-494 cells under unstimulated and ASC-CM (25% or 50%) stimulated conditions. Error bars show ±SEM and significant changes are represented as P-values (*p < 0.01, **p < 0.001). Exposure to ASC-CM increased both HIV-1 reactivation in U1 cells and HIV-1 LTR function in U-494 cells.

We compared the effect of adipose-derived MSCs (ASCs) and differentiated adipocytes (ADs) on virus production from latently-infected U1 monocytic cells, by measuring HIV-1 p24 levels in culture supernatants (Fig. 1). A representative image of ASCs (top) and oil red-O stained adipocytes (bottom) are shown in Fig. 1A. Culture conditioned medium (CM) were collected from these ASCs (ASC-CM) and adipocytes (AD-CM) and were then added to U1 cells at a 50% dilution. Bar graphs in Fig. 1B show HIV-1 p24 production by these U1 cells, following 3-, 5- and 7-days post-exposure to either ASC-CM or AD-CM. U1 growth media (U1-cont.), ASC growth media (ASC-cont.) and adipocyte differentiation media (AD-cont.) were used as controls. Exposure to U1-cont., ASC-cont. or AD-cont. media did not significantly alter HIV-1 production from U1 cells. However, exposure to ASC-CM or AD-CM caused a rapid and potent increase in HIV-1 p24 levels. Interestingly, ASC-CM caused a 6–10 fold higher HIV-1 p24 production by U1 cells as compared to those exposed to AD-CM. This indicated a crucial role of factors secreted by stem cells, and not differentiated adipocytes, in latency-reactivation. Next, we compared the effect of exposure to PMA (10 ng/mL) and/or ASC-CM (10% and 25%) on HIV-1 reactivation from U1 cells (Fig. 1C). Results showed that ASC-CM was as potent as PMA in increasing HIV-1 p24 production and coexposure to ASC-CM enhanced the latency reactivation efficacy of PMA. These observations suggest the therapeutic potential of ASC-CM when combined with current LRAs.

### Exposure to ASC-CM increased HIV-1 LTR directed reporter gene expression

We investigated whether latency-reactivation by ASC-CM occurs due to increased HIV-1 LTR directed gene expression. Studies were carried out using the U-494 cells, which were generated by stably transducing the U937 cell line with the lentivirus VRX-494 (Fig. 1D). This U-494 model enabled flow cytometric analysis of HIV-1 LTR function by measuring green fluorescent protein (GFP) expression after for 48 h of exposure to CMs. Exposure to ASC-cont. or AD-cont. media did not increase HIV-1 LTR activity. However, as compared to AD-CM, exposure to ASC-CM showed significantly (p < 0.01) higher LTR-directed GFP expression by the U-494 cells (Fig. 1E). Furthermore, exposure to increasing concentrations of ASC-CM (25% and 50%) showed a dose-dependent enhancement in the (a) number (Fig. 1F), (b) percentage (Fig. 1G) and (c) mean fluorescence intensities (MFI) of GFP-positive cells (Fig. 1H). Therefore, factors secreted by ASCs, can increase HIV-1 LTR-directed gene expression. These findings indicated that the latency-reactivation by the ASC-secreted factors may be due to increased LTR-directed transcription of the latent provirus.

### Latency-reactivation was corroborated using MSCs from both adipose-tissue (ASCs) and bone-marrow (BMSCs) obtained from multiple donors

**Figure 2:**
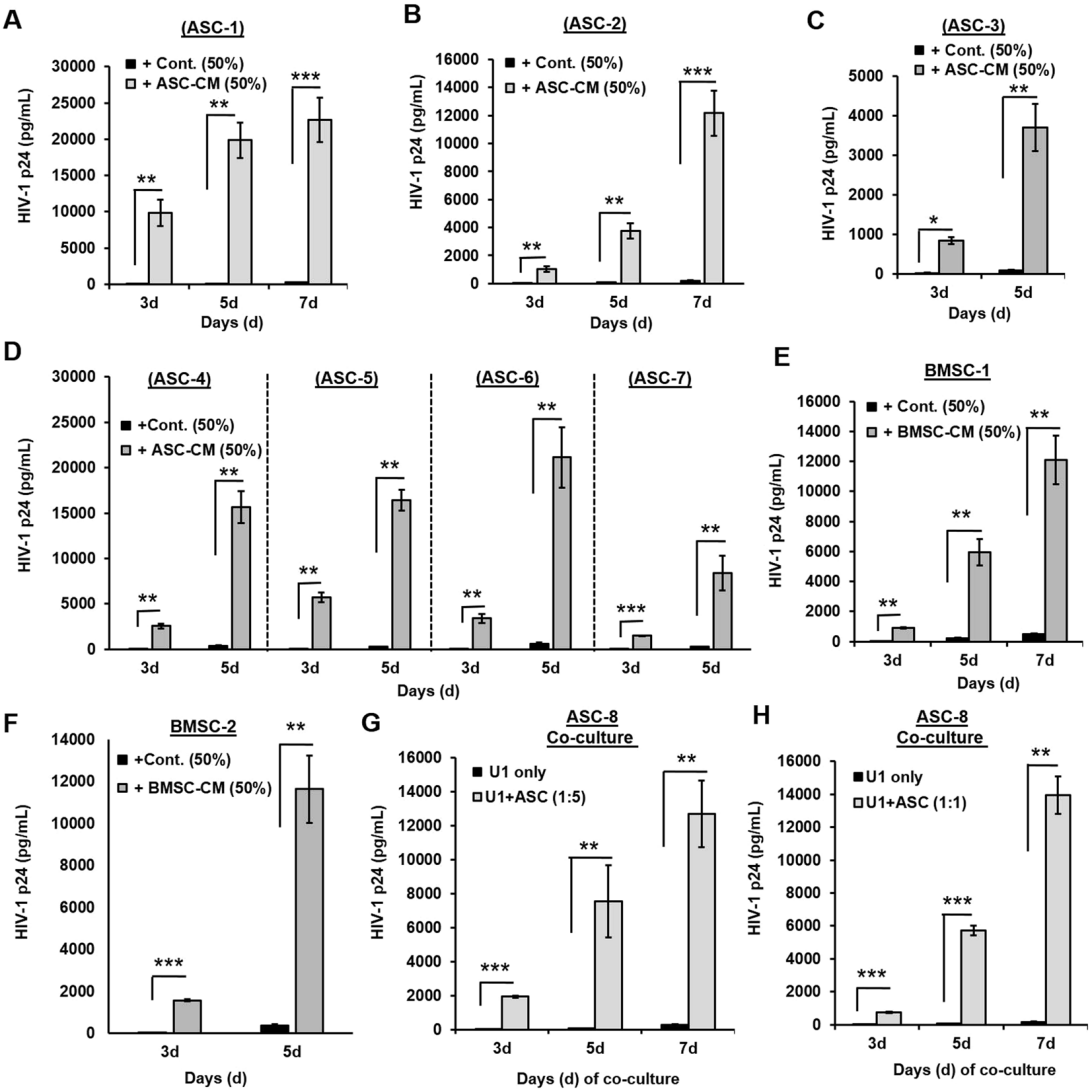
Latency-reactivation of U1 cells using both ASCs and BMSCs and in multiple donor batches. In (**A–D**), effect of CM from seven (7) different batches of adipose-tissue derived ASCs (ASC-1 to ASC-7). In (**E**,**F**), effect of CM from two (2) different batches of bone-marrow derived MSCs (BMSC) is shown. The latently-infected U1 cells were exposed to 50% ASC-CM or BMSC-CM and culture supernatants were collected after 3–7 days post-exposure to measure HIV-1 p24 levels by ELISA (pg/mL). U1 cells exposed to growth media only were used as background controls (Cont.). As compared to control media (black bars) U1 cells exposed to ASC-CM or BMSC-CM showed significant increases in HIV-1 p24 (gray bars). In (**G**,**H**), effect of ASC coculture with U1 cells is shown. For these studies, an eighth donor batch of ASCs (ASC-8) were co-cultured with U1 cells at different U1:ASC coculture ratios, either 1:5 (G) or 1:1 (H). Supernatants from U1 only cultures were used as control HIV-1 p24 values (black bars) and compared to culture supernatants after 3–7 days following ASC coculture (gray bars). Error bars show ± SEM and significant changes are represented as P-values (*p < 0.01, **p < 0.001, ***p < 0.0001). Potent latency-reactivation of U1 cells was corroborated using multiple ASC and BMSC batches, and using both CM and coculture experiments.

To fully corroborate the latency-reactivation potential of MSCs, we used stem cells obtained from eight more batches of adipose-derived MSCs (ASCs) and two different batches of bone-derived MSCs (BMSC) (Fig. 2). Experiments were carried out using both CM from MSCs (Fig. 2A–F) and following coculture with MSCs (Fig. 2G,H). A rapid increase in HIV-1 p24 production from U1 cells was clearly documented following exposure to factors secreted from ASCs (ASC-1 to ASC-7) and BMSCs (BMSC-1 and −2) (Fig. 2A–F). U1 cells that were exposed to control medium did not increase HIV-1 p24 production (solid black bars); however, in all cases, exposure to ASC-CM or BMSC-CM significantly increased HIV-1 p24 production within 3-days, which was further augmented at 5- and 7-days post-exposure. Next, we wanted to determine whether the coculturing of ASCs with U1 cells can similarly activate HIV-1 from latency. Indeed, using another more batche of ASCs (ASC-8) we confirmed the inductive effects of ASC coculture on U1 cells (Fig. 2G,H). Studies carried out at different U1:ASC cell coculture ratios (1:5 or 1:1) clearly showed that even low number of ASCs enable potent HIV-1 reactivation from U1 cells at 3-, 5- and 7-days post coculture. Therefore, both direct interaction between ASCs and U1 cells (coculture) and/or exposure to MSC-secreted factors (CM) are effective in latency reactivation. In addition, the latency-reactivation potential of MSCs may be a general phenomenon observed with stem cells from multiple donors.

### Exposure to factors secreted by latently-infected U1 cells were able to increase the migration and homing ability of ASCs

**Figure 3:**
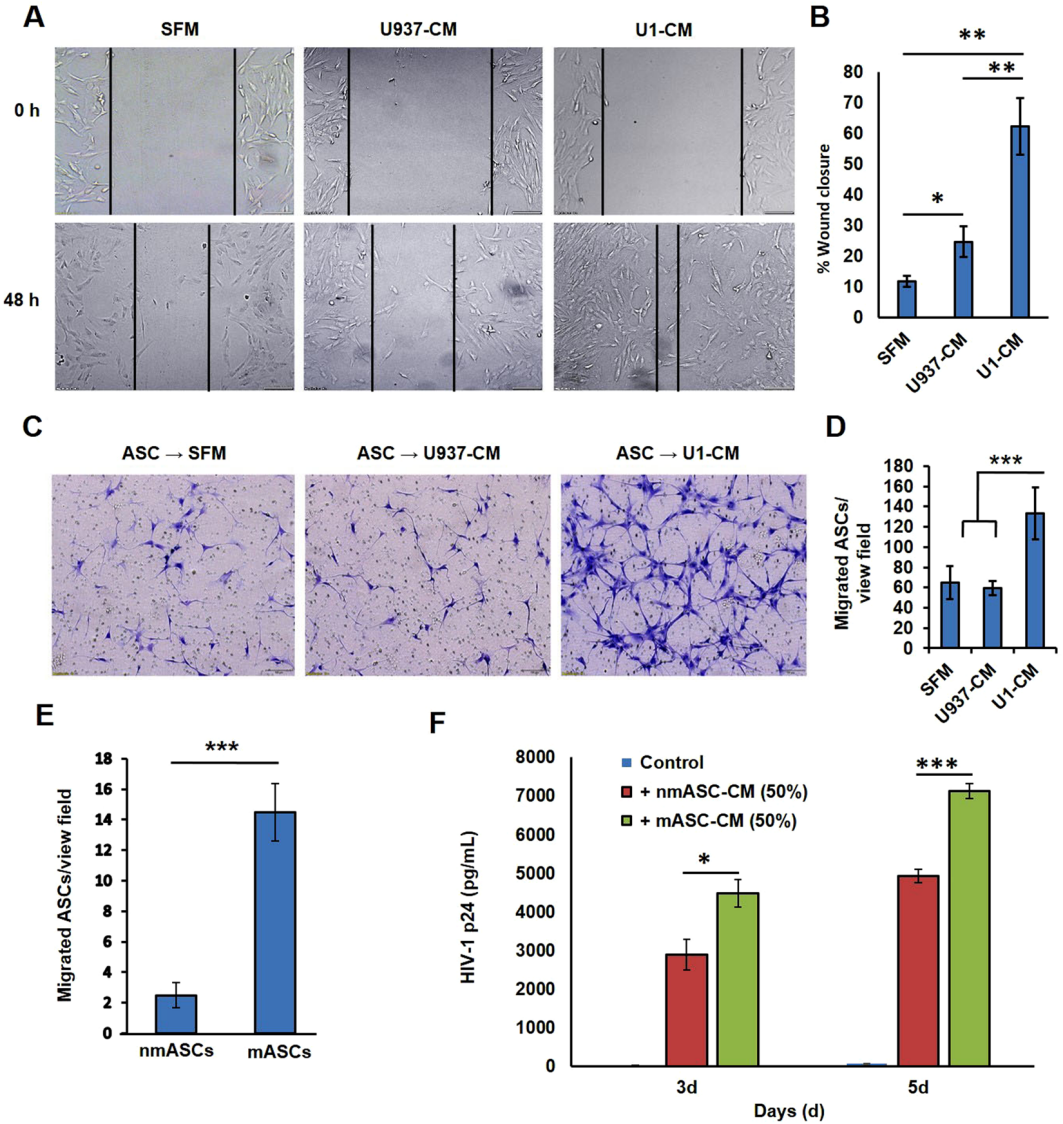
Effect of U1-secreted factor(s) on migration, homing and latency-reactivation potential of ASCs. (**A**,**B**) changes in ASC motility following exposure to 50% CM from either U937 or U1 cell (U937-CM and U1-CM, respectively), were measured by scratch-wound assays. Effect of serum free media (SFM) was used as control. (**A**) A representative image (10X magnification) of wounds at the initial time point (0 h) and at 48 h post-treatment. (**B**) Percent change in wound widths (closure) are shown in bar graphs (n = 3). Exposure to factors secreted by U1 cells increased the migratory ability of ASCs. (**C,D**) homing ability of ASCs towards either U937-CM or U1-CM was measured by transwell migration assays. (**C**) A representative image (10X) of ASCs attached to the bottom of transwell inserts (8 μm pore size). (**D**) Differences in the number of ASCs migrating in response to either SFM (control) or towards CMs from U937 or U1 cells, are shown in the bar graphs (n = 3). Exposure to factors secreted by U1 cells increased the homing ability of ASCs. In (**E,F**), a comparative analysis of homing ability (E) and latency-reactivation potential (F) of both the non-migratory ASCs (nmASC) and migratory ASCs (mASC) are shown. The nmASC and mASC were harvested from the top and bottom chambers, respectively. Each batch of ASCs was further propagated and CM collected from both subpopulations. **(E)** Shows differences in the homing ability of nmMSCs and mMSCs towards U1-CM. **(F)** Shows differences in the latency-reactivation potential of nmASC-CM and mASC-CM on U1 cells. The mASCs showed higher homing ability and retained their latency-reactivation potential. Error bars show ± SEM and significant changes are represented as P-values (*p < 0.01, **p < 0.001, ***p < 0.0001).

Studies were carried out to measure the effect of CM from U1 cells (U1-CM) on both ASC motility (Fig. 3A,B) and ASC homing (Fig. 3C,D), by using wound-heal assays and transwell migration assays, respectively. As compared to ASCs that were exposed to either serum free media (SFM) or factors secreted from uninfected U937 cells (U937-CM), exposure to the factors secreted from U1 cells (U1-CM) was able to significantly (p < 0.001) augment the migration of ASCs, as evident by decrease in wound width at 48 h post-exposure (Fig. 3A). We documented a significant decrease in the percent wound closure by ASCs following their exposure to U1-CM, as compared to SFM or U937-CM (Fig. 3B). Next, transwell migration assays were employed to investigate the effect of U1-CM on the attraction (homing) of ASCs towards the U1 cells (Fig. 3C). For these studies, ASCs were cultured in the top chamber of transwell plates and their migration to the bottom chamber was measured after 24 h. Light microscope images of ASCs attached to the bottom of the membrane inserts also indicated an activated phenotype of ASC that were exposed to U1-CM, as observed by the clustering of cells and their morphology. As compared to ASCs that transmigrated in response to SFM or U937-CM, exposure to U1-CM showed significantly (p < 0.0001) higher homing ability of ASCs (Fig. 3D). These findings clearly indicated that ASCs are attracted to factors secreted by the latently-infected U1 cells.

### The enriched migratory ASCs retained their reservoir cell-tropism and their latency-reactivation potential

Following the transwell migration of ASCs in response to U1-CM, these ASCs were harvested from both the upper and lower chambers to obtain the non-migratory ASCs (nmASC) and migratory ASCs (mASC), respectively. In subsequent transmigration assays, using both nmASCs and mASCs, we documented that the migration of mASCs towards the latently-infected U1-CM was 6–8 fold higher than the nmASCs (p < 0.0001) (Fig. 3E). Thus, findings showed that the mASCs retained their homing phenotype towards U1-CM. Next, we tested the HIV-1 reactivation potential of both nmASCs and mASCs. Thus, similar to the factors secreted by nmASCs (nmASC-CM), the mASC-CM was also able to increase virus production from U1 cells (Fig. 3F). Thus, ASCs possessing higher homing ability and latency-reactivation potential may be exploited towards a target reactivation of the latent HIV-1 reservoirs.

### ASCs are not productively infected but secrete factors that enable constant latency-reactivation of the U1 cells

**Figure 4:**
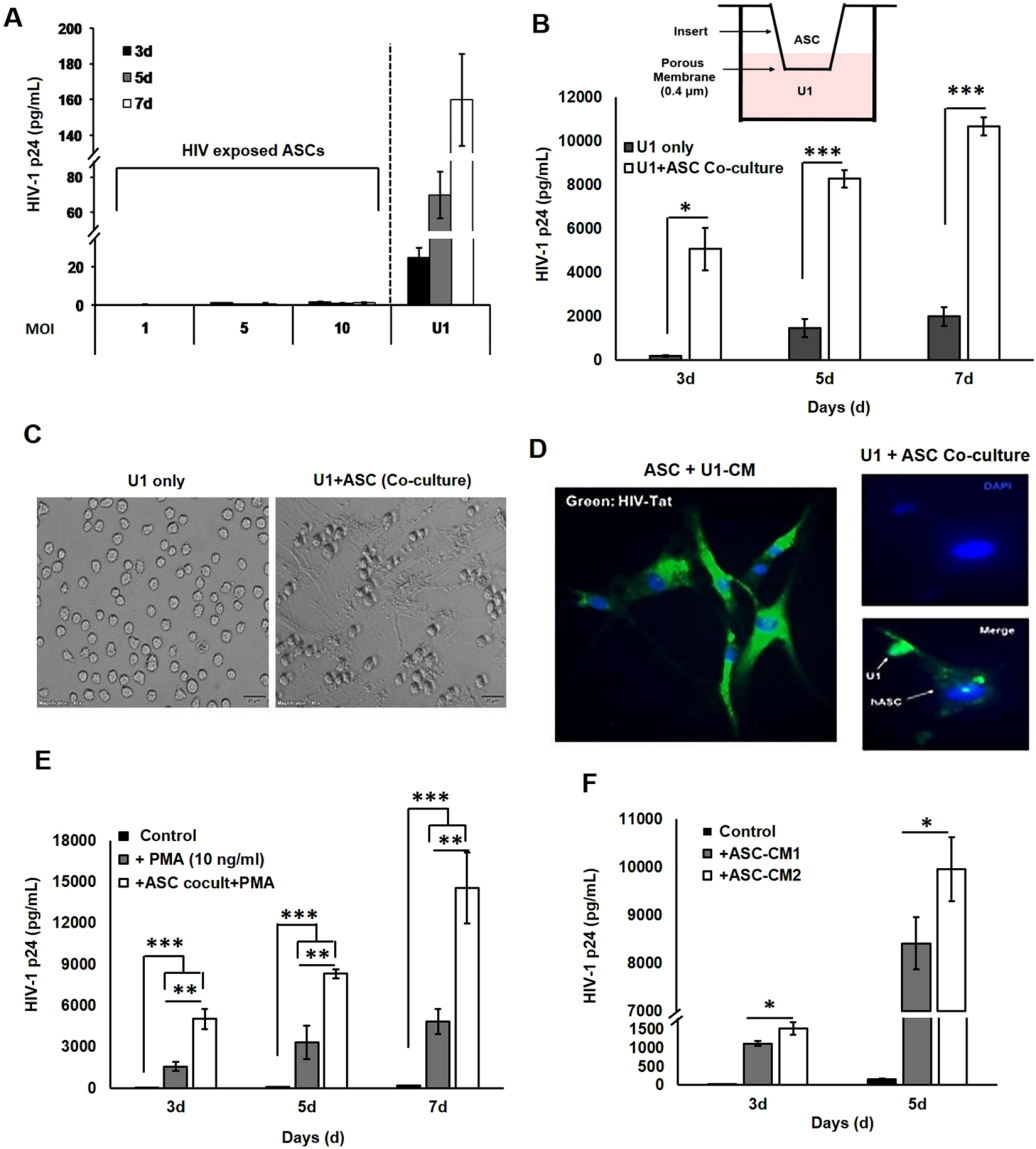
Cell-to-cell interactions between ASCs and U1, and its effects on the latency-reactivation potential of ASCs. (**A**) HIV-1 p24 production by ASCs exposed to high titer HIV-1 (maximum MOI = 10). Unstimulated U1 cells were used as controls. No detectable HIV-1 p24 was seen in culture supernatants from HIV-1 exposed ASCs. (**B**) Coculture assays using transwell chambers containing 0.4 μm membrane inserts enabled the monitoring of U1 reactivation following chronic exposure to ASC-secreted factors. ASCs were culture in upper chamber and U1 cells were cultured in the lower chamber (top panel). Culture media from the bottom chamber were collected after 3–7 days to measure HIV-1 p24 levels (pg/mL). A direct contact between both cell types is not necessary for ASC-mediated HIV-1 reactivation from U1 cells. (**C**) A representative photomicrograph of U1 cells alone (left) and U1 + ASC cocultures (right). Following 7-days of coculture, U1 cells preferentially adhered to the underlying ASCs. (**D**) Immunodetection of HIV-1 Tat protein in ASCs exposed to CM from PMA-stimulated U1 cells (left) and in direct coculture of ASCs with U1 cells (right). Merged images show Tat-specific staining (green) and Dapi-stained nuclei (blue). The Tat protein, secreted from U1 cells, is efficiently internalized by ASCs. (**E**) Effect of PMA-stimulation on ASC-mediated latency reactivation of U1 cells grown in direct contact with ASCs. Compared to unstimulated U1 cultures (black bars) the PMA-stimulated U1 cells showed increases in HIV-1 p24 levels (gray bars) which were significantly increased in ASC + U1 cocultures (white bars). (**F**) Effect of pre-exposure to U1-CM on the latency-reactivation ability of ASCs. For these studies, ASC-CMs were collected from both untreated ASCs (ASC-CM1) and from ASCs that were pre-primed by U1-CM (ASC-CM2). The latency-reactivation potential of both ASC-CM1 and ASC-CM2 were compared. Error bars show ±SEM and significant changes are represented as P-values (*p < 0.05, ***p < 0.0001). ASC interactions with U1 cells enable the uptake of Tat protein and ASCs pre-primed by U1-CM have increased latency-reactivation ability.

We previously documented that chronic exposure to HIV-1 (HTLV IIIB strain) enable latent infection of both BMSCs^29^ and ASCs^30^ and enabled the uptake of HIV-1 Tat protein by these cells. Therefore, here we wanted to first investigate whether acute exposure to HIV-1 (U1 strain) enables ASC infection and/or uptake of HIV-1 Tat protein, as well (Fig. 4). For these studies, ASCs were exposed to high titer virus (MOI = 10) from productively infected U1 cells (PMA stimulated). Next day, cells were thoroughly washed to remove any residual virus, cultured for 3–7 days, and supernatants were assessed for HIV-1 p24 production. In the unstimulated U1 cells, low level virus production (<200 pg/ml) was detected within 3-days, indicating their latent infection. However, in ASCs, exposure to HIV-1 did not show any detectable HIV-1 p24 production even after 7-days post-exposure (Fig. 4A). Thus, ASCs themselves are not susceptible to productive infection, and the rapid increase in HIV-1 following a single exposure to high dose ASC-CM (50%) may be primarily due to virus production from the U1 cells only. Therefore, next, we wanted to determine whether chronic exposure to factors secreted from a low number of ASCs is sufficient in increasing latency reactivation in U1 cells (Fig. 4B). This was tested using a transwell coculture chamber containing a 0.4 μm pore insert, which enabled the transfer of media components only, but no cells transmigrated. The ASCs were cultured on the top chamber and U1 cells were cultured in the bottom chamber of transwell plates (Fig. 4B, top). Results showed that the ASC-secreted soluble factors are able to reactivate HIV-1 from U1 cells, and this was clearly evident after 3-, 5- and 7-days post transwell coculture (Fig. 4B, bottom). Thus, soluble factors from ASCs enable productive infection in the latently infected U1 cells.

### U1 cells cocultured with ASCs enabled the transfer of HIV-1 Tat protein and activated ASCs showed higher latency-reactivation ability

Tat protein is secreted by HIV-1 infected cells, and its uptake can activate signaling in neighboring uninfected cells^31,32^. Direct coculture experiments showed that the latently-infected U1 cells interact closely with ASCs (Fig. 4C) and this enabled the transfer of Tat protein from U1 cells to the ASCs (Fig. 4D). Images show that, when cultured without ASCs, these U1 cells grew as individual floating cells (Fig. 4C, left panel). However, following 7-days of coculture with ASCs, these U1 cells were attached to the underlying ASCs and grew in clusters (Fig. 4C, right). In subsequent experiments, immunofluorescence microscopy (IFM) of ASCs, that were either exposed to CM from PMA-stimulated U1 cells (Fig. 4D, left) or co-cultured with unstimulated U1 cells (Fig. 4D, right) clearly showed Tat-specific immunostaining (green) in ASCs exposed to PMA-activated U1-CM. In addition, Tat protein was detected in both the ASC-activated U1 cells and in the U1-associated ASCs. Therefore, we measured virus production by U1 cells following their direct coculture with ASCs (1:1 cell ratio) (Fig. 4E). Similar to the effect of ASC-CM, direct contact with ASCs increased HIV-1 p24 production at 3, 5 and 7 days post coculture. Most interestingly, similar to the data obtained with ASC-CM and PMA costimulation, we observed a significantly (p < 0.001) higher latency-reactivation when the U1:ASC cocultures were also exposed to PMA. Thus, both ASCs and U1 cells are activated within inflammatory microenvironments to enable significant latency-reactivation. Next, we investigated whether the latency-reactivation ability of ASCs can be increased following their exposure to factors from U1 cells (Fig. 4F). For these studies, CMs were collected from both unstimulated ASCs (ASC-CM1) and from ASCs that were pre-stimulated with U1-CM (ASC-CM2). We compared the effect of ASC-CM1 and ASC-CM2 on HIV-1 p24 production by the U1 cells. Results clearly showed that the ASCs that were pre-stimulated with U1-CM secreted factors that enabled significantly higher latency-reactivation (p < 0.05).

### Latency-reactivation by MSCs was corroborated in two T-cell line models of HIV-1 latency

**Figure 5:**
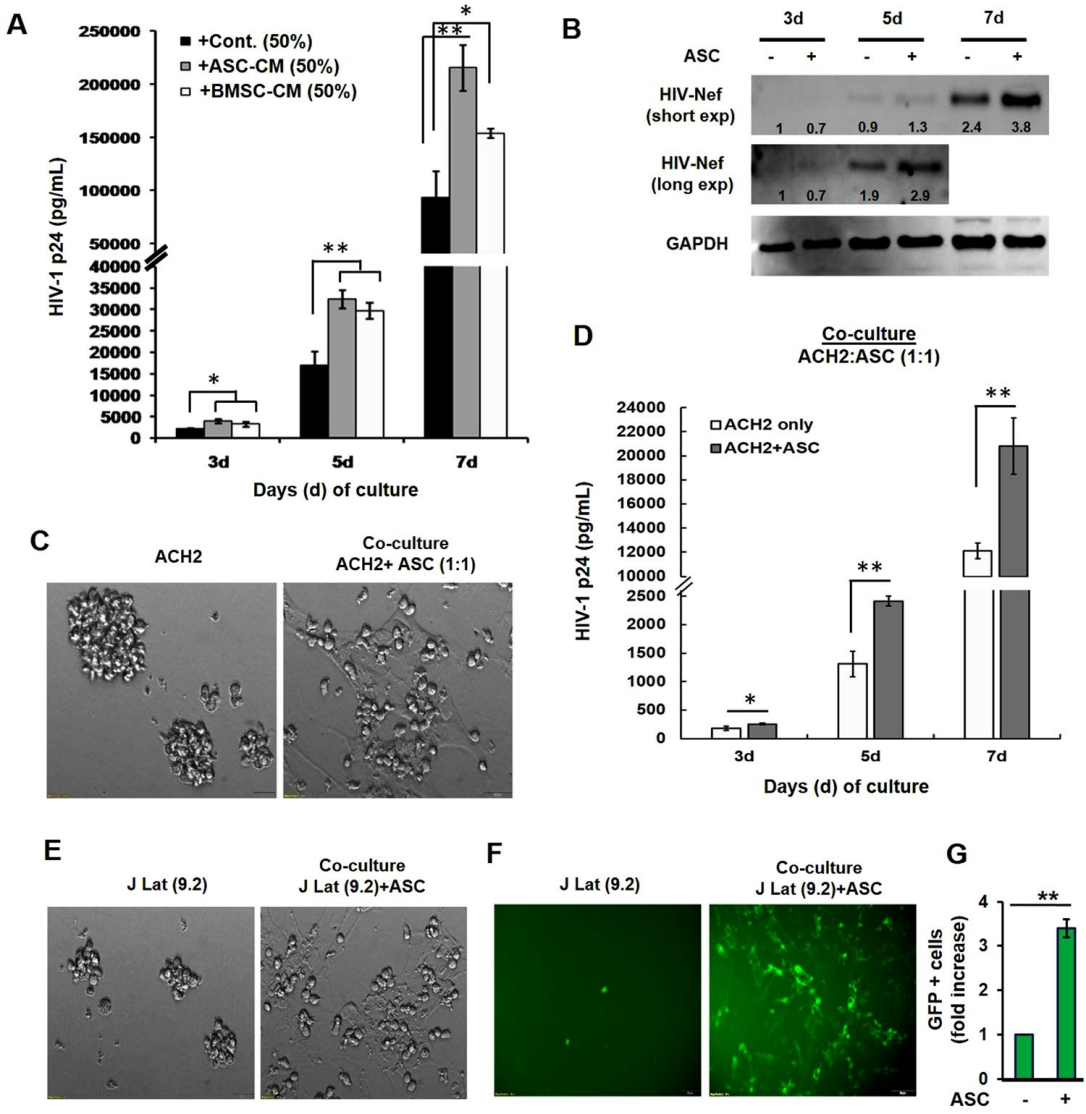
Effect of MSC-secreted factors on HIV-1 reactivation from T-cells. We investigated whether MSCs can reactivate HIV-1 using two well-established T-cell line models, i.e. ACH2 and J-Lat (9.2). (**A**) Effect of exposure to ASC-CM or BMSC-CM on virus reactivation from ACH2 cells. As compared to unstimulated ACH2 cells (black bars) exposure to 50% CM from both ASCs (gray bars) and BMSCs (white bars) increased HIV-1 p24 production (pg/mL) at 3, 5 and 7-days. (**B**) Western immunoblot analysis of lysates from the ACH2 cells cocultured with ASCs was further confirmed HIV-1 activation, where increased expression of HIV-1 Nef protein was clearly evident. Relative band intensity (presented the numbers to the corresponding lanes) was compared by the Image-J software (version 1.50). (**C**) A representative image of ASCs cocultured with ACH2 cells. Following 7-days of coculture, unlike the ACH2 cultures alone (left) the cocultured ACH2 cells preferentially adhered to the underlying ASCs (right). (**D**) As compared to ACH2 cells alone, significantly higher HIV-1 p24 production was observed when ACH2 cells were cocultured with ASCs. (**E**) ASC-mediated latency reactivation was investigated using the J-Lat (9.2) cell line, which expresses GFP from an integrated HIV-1 provirus. In coculture experiments, the J-Lat (9.2) cells were found to directly interact with ASCs (right) as compared to themselves (left). (**F**) A representative image of GFP expression in J-Lat (9.2) cells, alone (left) or following ASC coculture (right). (**G**) Fold change in GFP-positive J-Lat (9.2) cells, in the absence or presence of ASCs. Error bars show ±SEM and significant changes are represented as P-values (*p < 0.01, **p < 0.001). The latency-reactivation potential of ASCs was corroborated using two established T-cell line models of HIV-1 latency.

We investigated whether MSCs can reactivate the HIV-1 provirus in other latently infected cells by using two well-established T-cell line models, i.e. ACH2 and J-Lat(9.2) (Fig. 5). Exposure to CM from ASCs (ASC-CM) or BMSCs (BMSC-CM) caused a temporal increase in HIV-1 p24 production in ACH2 cell cultures (Fig. 5A). This latency-reactivation was further confirmed by Western immunoblot (IB), where increased expression of HIV-1 Nef protein was clearly evident in lysates from ASC exposed ACH2 cells (Fig. 5B). Next, we measured the effect of ASC coculture with ACH2 cells (Fig. 5C,D). Similar to the U1 cells, coculture studies showed that the ACH2 cells directly interacted with ASCs (Fig. 5C) and significantly higher HIV-1 p24 production was documented when ACH2 cells were cocultured with ASCs (Fig. 5D). In subsequent studies, ASC-mediated latency reactivation was also investigated using the J-Lat (9.2) cell line. In these cells, a GFP open reading frame (ORF) had been inserted within the ENV gene, which enables a direct measurement of LTR-directed provirus reactivation by GFP fluorescence. In coculture experiments, we observed that the J-Lat (9.2) cells strongly attached to the ASCs (Fig. 5E) and this enabled significant increases in reporter expression, as evident by increases in both GFP fluorescence (Fig. 5F) and fold increase in GFP-positive cells (Fig. 5G). Thus, findings using both U1 and ACH2 latency models clearly demonstrated that the latency-reactivation potential of ASCs is a phenomenon associated with both monocytic and T-cells. In addition, findings using U937-VRX and J-Lat (9.2) models implicated a crucial role for ASC-secreted factors in activating the latent provirus and HIV-1 LTR directed gene expression. Therefore, subsequent studies were carried out to investigate: (a) the therapeutic potential of ASC-CM in combination with LRAs; (b) the molecular mechanism(s) associated with latency-reactivation.

### Coexposure to ASC-CM enhanced the latency-reactivation ability of panobinostat

**Figure 6:**
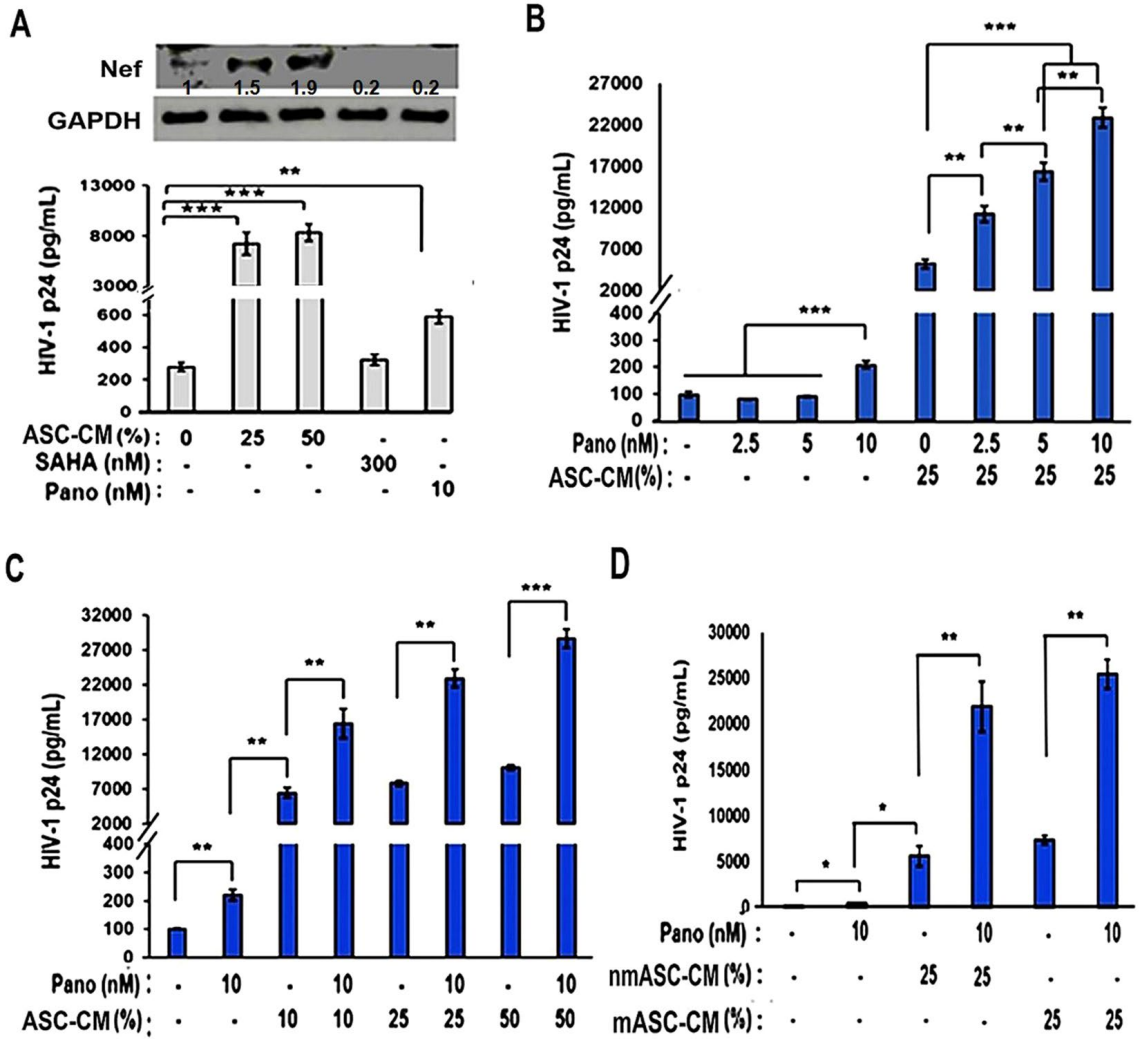
Effect of ASC-CM on latency-reactivation efficacy of panobinostat. (**A**) Effect of exposure to ASC-CM (25% or 50%) or HDACi [SAHA or Panobinostat(Pano)] on HIV-1 Nef protein expression (top panels) and HIV-1 p24 production (bottom panels) by the U1 cells. Relative Western blot band intensity (presented the numbers to the corresponding lanes on the top panel) was compared by the Image-J software (version 1.50). The ASC-CM mediated latency reactivation was much more potent, as compared to SAHA (300 nM) or panobinostat (10 nM). (**B**) Effect of ASC-CM (25%) coexposure on panobinostat (2.5–10 nM) mediated increases in HIV-1 p24 production by U1 cells. Coexposure to ASC-CM increased the latency-reactivation efficacy of panobinostat. (**C**) Effect of increasing concentrations of ASC-CM (10–50%) on panobinostat (10 nM) induced latency-reactivation. Coexposure to even low concentrations of ASC-CM increased latency-reactivation by panobinostat. (**D**) Effect of CM, from either non-migratory or migratory ASCs (nmASCs and mASCs, respectively) on latency-reactivation by panobinostat (10 nM). The mASCs retain their ability to activate HIV-1 p24 from U1 cells and secrete factors that augment the efficacy of panobinostat. Error bars show ± SEM and significant changes are represented as P-values (*p < 0.01, **p < 0.001, ***p < 0.0001). Coexposure to ASC-secreted factors potentiate the efficacy of a clinically approved HDACi, i.e. panobinostat (Pano).

Several class-I HDAC inhibitors, such as vorinostat and panobinostat, are being clinically tested as epigenetic activators of the latent HIV-1 provirus^33,34^. However, studies have also questioned their efficacy in HIV-1 reservoirs *in vivo*^35,36^. Therefore, recent publications have underscored the need for using different combinations of LRAs to enable potent latency-reactivation^37,38^. We investigated whether coexposure to the ASC-CM can increase the LRA efficacy of vorinostat (SAHA) and panobinostat (Pano) (Fig. 6). At physiologically achievable concentrations of panobinostat (10 nM) or vorinostat (300 nM) we observed only a 2–3 fold increase in HIV-1 p24 production from U1 cells (Fig. 6A, bottom panel) and no detectable increase in HIV-1 Nef protein was observed (Fig. 6A, top panel). However, exposure to ASC-CM (25%) showed a 20–25 fold increase in both HIV-1 p24 production (Fig. 6A, bottom panel) and clearly increased Nef protein expression in the U1 cells (Fig. 6A, top panel). Findings demonstrated that, as compared to either vorinostat or panobinostat, factors secreted by ASCs are significantly more effective in latency-reactivation. Next, we investigated whether costimulation with ASC-CM can increase the LRA efficacy of panobinostat. Indeed, compared to increasing doses of panobinostat alone (Pano; 2.5–10 nM) coexposure to ASC-CM (25%) enabled significantly higher HIV-1 p24 production from U1 cells (Fig. 6B). Furthermore, coexposure to increasing doses of ASC-CM (10–50%) enabled significantly higher HIV-1 p24 production from U1 cells that were treated with panobinostat (10 nM) (Fig. 6C). Since our previous findings using the ASC subpopulation which preferentially migrated to U1 cells (mASCs) showed their latency-reactivation potential, we wanted to investigate whether mASC-CM can also increase the therapeutic potential of panobinostat. Similar to nmASC-CM, costimulation with mASC-CM (25%) significantly enhanced the LRA efficacy of panobinostat (10 nM) (Fig. 6D). Above findings implicated the therapeutic potential of reservoir-tropic ASCs in increasing the efficacy of clinically approved LRA drugs such as panobinostat.

### ASC-mediated latency-reactivation in U1 cells may exploit a non-canonical PI3K-NFκB signaling pathway

**Figure 7:**
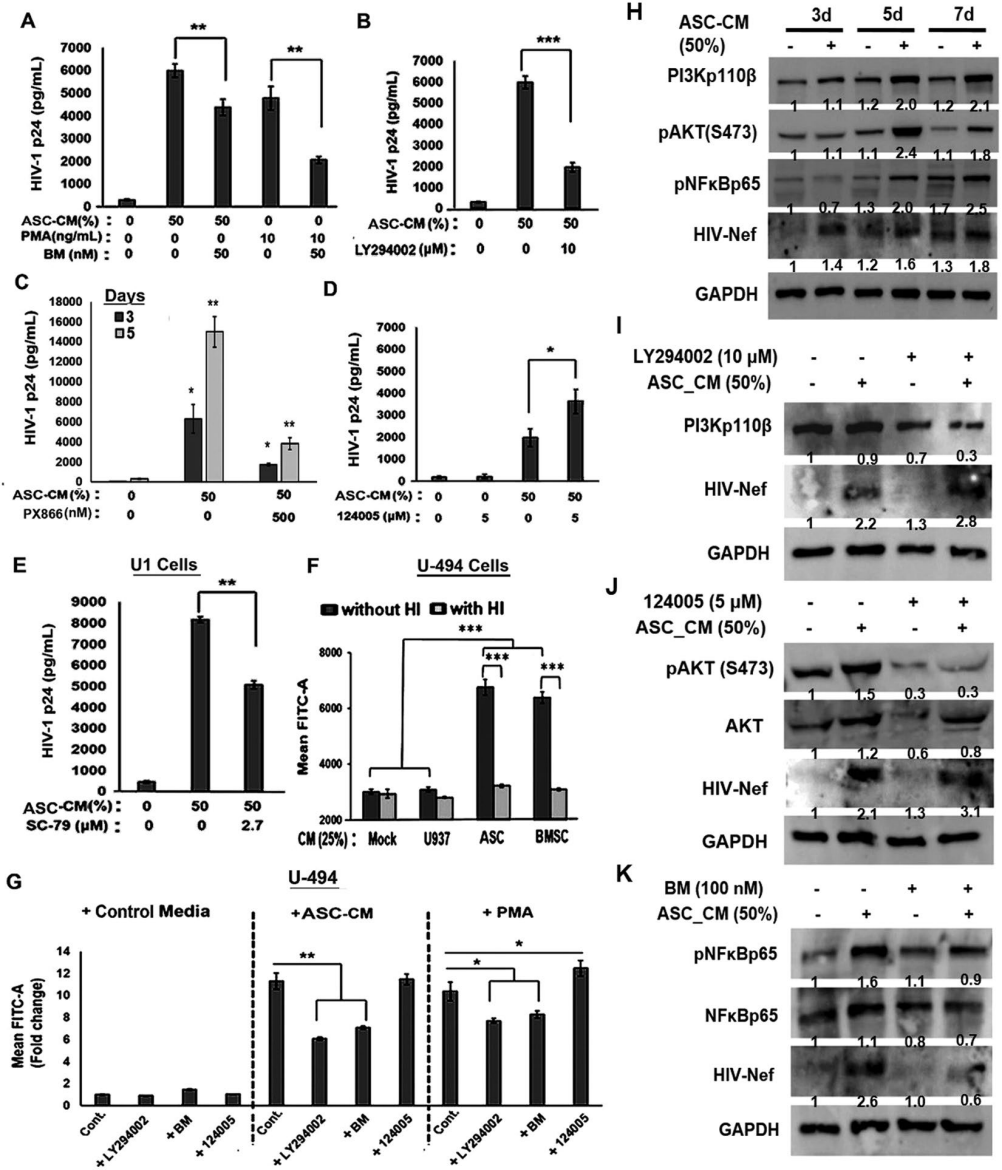
Role of NF-κB, PI3K and AKT pathways in ASC-mediated latency-reactivation of U1 cells. (**A**) Effect of NF-κB inhibitor, bardoxolone-methyl (BM; a.k.a. CDDO-Me) on HIV-1 p24 production by U1 cells, stimulated with either ASC-CM (50%) or PMA (10 ng/mL). Exposure to BM (50 nM) decreased HIV-1 p24 production in both PMA-induced and ASC-CM-stimulated U1 cells. (**B,C**) effect of PI3K inhibitors, (**B**) LY294002 (10 μM) or (**C**) PX866 (500 nM) on HIV-1 p24 production in ASC-CM (50%) stimulated U1 cells. PI3K inhibitors suppressed latency-reactivation by ASC-CM. (**D,E**) effects of AKT inhibition by 124005 (5 μM) and AKT activation by SC-79 (2.5 μM) on ASC-CM (50%) induced HIV-1 p24 production. Latency-reactivation by ASC-CM is increased following AKT inhibition and decreased following AKT induction. (**F**) Effect of heat-inactivation (HI) of ASC-CM or BMSC-CM on HIV-1 LTR-directed GFP expression (MFI) by the U-494 cells. Heat labile factors in CMs are responsible for LTR activation. (**G**) Effect of PI3K and NF-κB inhibitors on HIV-1 LTR function in U-494 cells, stimulated with either ASC-CM (50%) or PMA (10 ng/mL). GFP expression (MFI) was monitored after 48 h and fold change (compared to control media) are shown. Both LY294002 (PI3K inhibitor) and BM (NFκB inhibitor) suppressed HIV-1 LTR function, but 124005 (AKT inhibitor) showed an inductive effect. Error bars show ± SEM and significant changes are represented as P-values (*p < 0.01, **p < 0.001, ***p < 0.0001). (**H**) Immunoblot (IB) analysis of HIV-1 Nef, PI3K(p110β), and phosphorylated AKT [pAKT(473)] and NFκB [pNFκBp65] proteins in unstimulated and ASC-CM (50%) stimulated U1 cells. GAPDH was used as a loading control. Exposure to ASC-CM causes temporal increases (3–7 days) in PI3K, NFκB and AKT activation, and augments HIV-1 Nef expression. Effects of LY294002 (**I**), 124005 (**J**) and BM (**K**) on ASC-CM (50%) stimulated HIV-1 Nef, PI3K(p110β), and both total & activated forms of AKT and NFκBp65 proteins at 24 h post-stimulation. Exposure to inhibitors suppressed PI3K(p110β), pNFκBp65 and pAKT(S473) levels. HIV-1 Nef in ASC-CM treated U1 cells was also decreased following exposure to signal transduction inhibitors. Relative band intensity (presented the numbers to the corresponding lanes) was compared by the Image-J software (version 1.50). The NF-kB, PI3K and AKT pathways play crucial roles in regulating HIV-1 reactivation in U1 cells and HIV-1 LTR activation in U-494 cells.

By using specific signal transduction inhibitors, we carried out studies to delineate the signaling pathways involved in ASC-mediated latency-reactivation in U1 cells (Fig. 7). The NF-κB transcription factor is a primary mechanism of HIV-1 reactivation from latency. Therefore, we first tested whether pre-exposure to a potent NF-κB inhibitor, CDDO-Me (bardoxolone-methyl; BM) can suppress HIV-1 p24 production from U1 cells, which were stimulated with either PMA (10 ng/mL) or ASC-CM (50%) (Fig. 7A). NF-κB inhibition suppressed PMA-stimulated HIV-1 p24 production by more than 50% (p < 0.001) but only suppressed ASC-mediated inductive effects by approximately 25%. Thus, factors secreted by ASCs (ASC-CM) may utilize signaling mechanisms other than NFκB to activate viral latency in U1 cells. Therefore, we tested the effects of two PI3K pathway inhibitors, LY294002 and PX-866. Pre-exposure to LY294002 (10 μM) suppressed the inductive effects of ASC-CM by more than 70% (p < 0.0001) (Fig. 7B). Importantly, a more potent and clinically tested PI3K inhibitor, PX-866 was also effective in decreasing ASC-CM-mediated effects. As much as a 70–85% decrease in ASC-CM induced HIV-1 p24 production was observed even with a very low dose of PX-866 (500 nM) (Fig. 7C). Therefore, finding indicated a role for both PI3K and NFκB signaling pathways in regulating the ASC-CM mediated latency-reactivation.

The PI3K signaling cascade activates the downstream AKT pathway^39^. Therefore, we investigated the effects of AKT-inhibition (Fig. 7D) or AKT-induction (Fig. 7E) on ASC-CM mediated latency-reactivation. Surprisingly, in contrast to the potent suppressive effects of the PI3K inhibitors (LY294002 & PX-866), pretreatment of U1 cells with the AKT-inhibitor, 124005 significantly increased HIV-1 p24 production (Fig. 7D). Furthermore, the AKT-inducer, SC-79 significantly decreased HIV-1 p24 production (Fig. 7E). Therefore, although the PI3K pathway is associated with latency-reactivation, the downstream AKT pathway may have a suppressive effect. Thus, ASCs reactivate HIV-1 from U1 cells via a non-canonical PI3K and NFκB dependent mechanism, which is negatively affected by AKT.

### ASC-mediated increases in HIV-1 LTR function in U-494 cells occurred via a PI3K & NFκB-dependent mechanism

We investigated whether the HIV-1 inductive effects of ASCs occur due to HIV-1 LTR activation via the PI3K and NKκB signaling pathways. Studies were carried out using the LTR-GFP reporter cell line, U-494 (Fig. 7F,G). Exposure to control MSC-CM, either ASC-CM or BMSC-CM, were able to increase HIV-1 LTR directed GFP expression; however, exposure to the heat-inactivated (HI) ASC-CM or BMSC-CM did not increase LTR function (Fig. 7F). Therefore, heat-labile soluble factors were found to be responsible for increased LTR function. Next, we used different signal transduction inhibitors and assessed their ability to suppress GFP levels in U-494 cells (Fig. 7G). Increased HIV-1 LTR function, seen with both ASC-CM and PMA stimulation, was inhibited by the NFκB inhibitor CDDO-me (BM; 50 nM) and the PI3K inhibitor, LY294002 (10 μM). Interestingly however, exposure to the AKT-inhibitor (124005; 5 μM) did not significantly increase HIV-1 LTR function. Thus, similar to the activation of infectious HIV-1 in U1 cells, the activation of HIV-1 LTR in U-494 cells require the activation of both PI3K and NFκB pathways.

### PI3K(p110β) and NFκB(p65) activation correlated with ASM-CM mediated latency-reactivation and increased HIV-1 Nef protein levels

To measure the activation of above signaling pathways, we carried out Western immunoblot analysis of PI3K(p110β) and both total and phosphorylated forms of AKT (S473) and NFκB(p65) proteins using lysates from both unstimulated and ASC-CM- stimulated U1 cells. In cells that were exposed to ASC-CM (50%) for 3, 5 and 7 days, we observed that HIV-1 Nef protein levels are increased within 3-days post-exposure to ASC-CM and Nef levels were further enhanced at 5 and 7 days (Fig. 7H). Similarly, we observed temporal increases in PI3K(p110β), pAKT(S473) and pNFκB(p65) in the ASC-CM-stimulated U1 cells. Both PI3K(p110β) and pAKT levels were significantly higher at the 3-day and 5-day time points and showed a slight decrease at the 7-day time point. Increased pNFκB(p65) levels were also clearly evident. This indicated that the above three signaling pathways are activated in U1 cells and may be directly involved in latency-reactivation, and increased HIV-1 Nef protein expression. Next, we tested whether specific inhibitors of these three signaling pathways can suppress ASC-CM stimulated Nef expression (Fig. 7I–K). For these experiments, U1 cells were pre-exposed to the inhibitors for 2 h prior to their stimulation with ASC-CM (50%) and cells were harvested after 24 h. Western immunoblot analysis demonstrated that HIV-1 Nef levels were significantly increased following 24 h of ASC-CM exposure. Although PI3K(p110β) was increased at the later time points, a similar increase in PI3K(p110β) was not seen after 24 h stimulation (Fig. 7I). However, both pAKT(S473) (Fig. 7J) and pNFκB(p65) (Fig. 7K) levels were detectably increased and slight increases in total AKT and pNFκBp65 were also seen at this time point. In addition, IB studies using cells pre-exposed to the AKT-inhibitor, 124005 (5 μM) or the NFκB inhibitor, BM (100 nM) clearly showed the specificity of these inhibitors. Both pAKT(S473) (Fig. 7J) and pNFκB(p65) (Fig. 7K) levels were decreased, and these inhibitors were able to suppress the ASC-CM mediated increase in HIV-1 Nef expression. Most interestingly, even low dose (100 nM) of BM was sufficient in suppressing Nef protein activation. Above IB studies confirmed that exposure to ASC-CM activates PI3K, AKT and NFκB signaling pathways, and a cross-talk between these pathways may be involved in regulating latency and/or reactivation of HIV-1 in the U1 cells.

## Discussion

**Figure 8:**
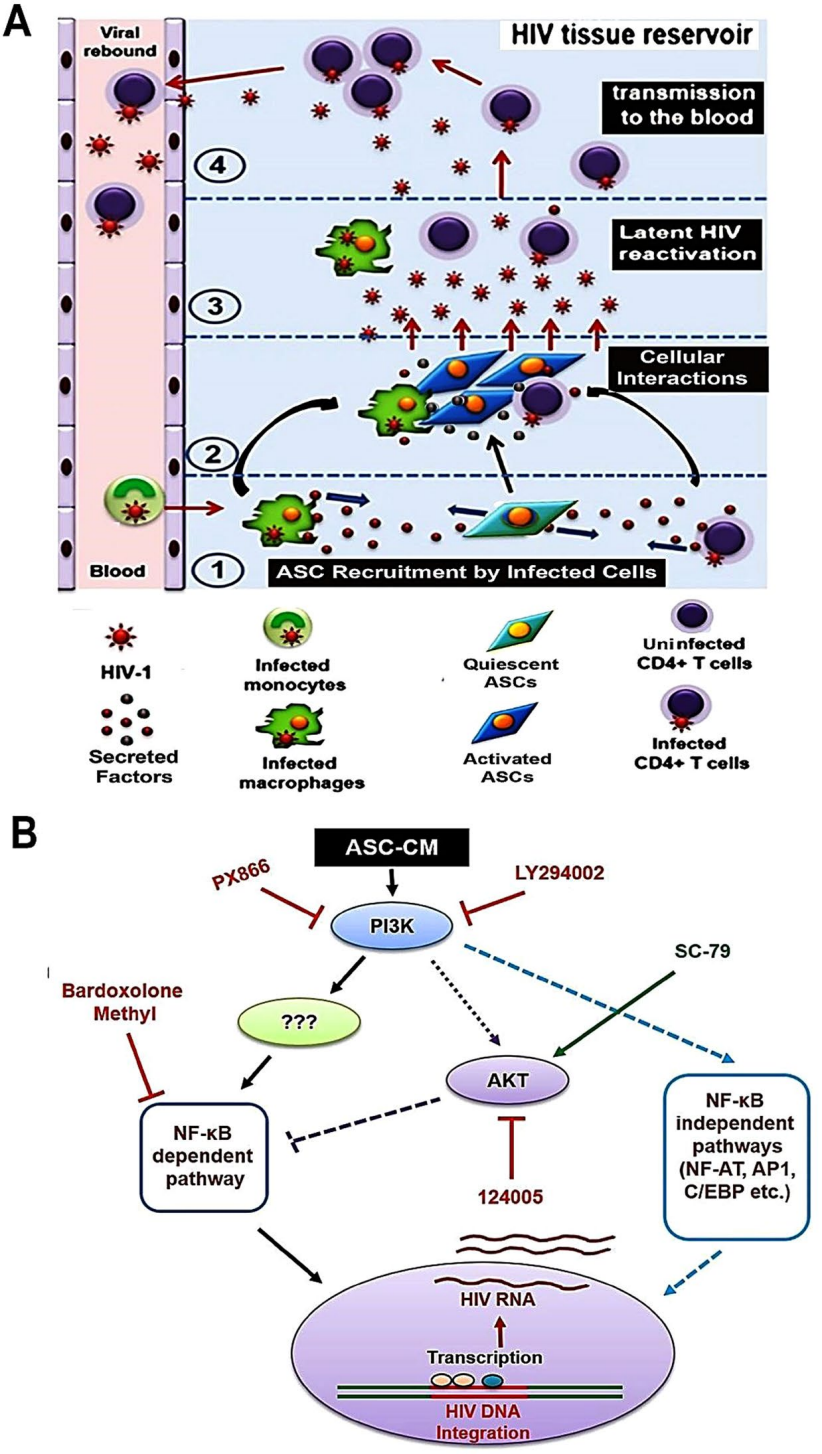
Putative molecular effects of MSC-secretome on latency-reactivation from tissue HIV-1 reservoirs. (**A**) Schematics of possible interactions between MSCs & latently-infected cells within tissue HIV-1 reservoirs. The following steps may be involved in persistence, propagation and transmission of HIV-1 from reservoirs microenvironments: (1) Factors secreted from latently-infected cells (MACs & THLs) may recruit tissue-resident MSCs; (2) these reservoir-recruited MSCs may closely interact with latently-infected cells, where reciprocal signaling (cell-to-cell and/or secretome-mediated) may facilitate virus reactivation; (3) the reservoir-recruited MSCs may also be activated to secrete factors that constantly reactivate HIV-1 from latency, generating an inflammatory microenvironment that enable further recruitment of infectable cells; and (4) these productively infected reservoir cells may then migrate into the blood and enable rapid viral rebound. (**B**) Signaling pathways involved in MSC-secretome (ASC-CM) mediated HIV-1 reactivation from latently-infected MACs (U1 cells). The potent suppressive effects of LY294002 & PX-866 implicate a direct role for the PI3K signaling pathway. The potent effect of Bardoxolone-Methyl (CDDO-Me) also suggests that NF-κB activation is a mechanism of this latency-reactivation. Interestingly however, increased reactivation by the AKT inhibitor (124005) and decreased reactivation by the AKT activator (SC-79) also suggest the involvement of a non-canonical PI3K-NFκB dependent pathway, where the downstream AKT pathway has a negative regulatory effect on MSC-mediated latency-reactivation from tissue resident MACs.

In HIV-1 positive patients on HAART, low level viral replication continues to occur within tissue HIV-1 reservoirs, where subtherapeutic drug concentrations facilitate the selection of resistant mutants^4,5^. The confirmation of active virus production from latent reservoirs was first documented by Chun *et al*., (2000) where they showed that the genotype of virus that rebounds following HAART discontinuation are distinct from those present in the blood^40^. Indeed, prompt viral rebound from reservoirs further corroborates that low-level viral replication continues to occur despite HAART^4,7^. Multiple studies have shown that latently-infected cells are major players in HIV-1 persistence, propagation and dissemination^16,41–43^. However, systemic HAART is primarily focused on decreasing plasma viral load and does not target the latently-infected cells in anatomical reservoirs^12,17,33,35^. Factors that critically regulate latency and/or reactivation of HIV-1 within reservoir microenvironments are not well understood. MSCs within lymphoid tissues are well-known to interact with both macrophages and T-cells to regulate their activation phenotype^24,27,28^. Our current findings, using the latently-infected monocytic cell line (U1) and the T-cell lines [ACH2 and J-Lat (9.2)], clearly indicated a novel role of tissue resident MSCs in dictating latency-reactivation. Furthermore, our molecular mechanistic studies implicate a crucial role of PI3K and NFκB signaling pathways, and the potential of several pharmaceutical drugs to suppress constant reservoir reactivation by MSCs. Last, but not the least, we provide evidence that factors secreted from MSCs, or the novel signaling pathways activated during latency-reactivation, may be exploited to enhance the latency-reactivation efficacy of approved LRAs like panobinostat. Therefore, MSCs may have a crucial function in dictating HIV-1 reservoir persistence & propagation (Fig. 8A) and a clear understanding of signaling pathways that regulate MSC-mediated latency-reactivation may provide effective therapeutic strategies against these reservoirs (Fig. 8B).

We observed that factors secreted by MSCs enable HIV-1 reactivation from two latently-infected cell line models, i.e. U1 (monocytic cells) and ACH2 (T-cells). Factors secreted from MSCs (both ASCs and BMSCs) but not from adipocytes, enabled rapid and potent HIV-1 activation from both U1 cells (Figs 1,2) and ACH2 cells (Fig. 5). Studies using both U-494 cells (Figs 1,7) and J-Lat (9.2) cells (Fig. 5) indicated that soluble factors from MSCs can increase HIV-1 LTR directed transcription of the latent provirus. Furthermore, we documented that the latently-infected cells secrete factors that efficiently attract MSCs (Fig. 3) and MSCs with enhanced migratory ability (mMSCs) retain their latency-reactivation ability (Fig. 3). Importantly, coexposure to the MSC-secreted factors enhanced the LRA efficacy of panobinostat (Fig. 6). Studies using signal transduction inhibitors implicated a crucial role for both the PI3K and NFκB signaling pathway in ASC-mediated latency-reactivation. The PI3K inhibitor, PX-866 and the NFκB inhibitor, CDDO-me (BM) two drugs currently in several clinical trials, were able to suppress the HIV-1 reactivation by the MSC secreted factors. Thus, latently-infected cells can recruit MSCs which enable latency-reactivation via a PI3K & NFκB dependent mechanism.

Although we did not investigate the effects of ASC-secreted factor(s) on primary cells, findings using the well-established U1 cell line model^44,45^ and the two T-cell line models of HIV-1 latency, i.e. ACH2 and J-Lat (9.2)^13–15^ clearly indicate a general phenomenon that stromal factors are directly involved in latency-reactivation. Furthermore, the latency-reactivation potential of both ASCs and BMSCs suggest their crucial role in latency-reactivation within multiple tissue HIV-1 reservoirs.

We documented that soluble factor(s) secreted from latently-infected cells enable MSC activation, and pre-incubation of MSCs to U1 secreted factors further increased the migration and homing abilities of MSCs. Indeed, several recent investigations have provided evidence that the secretome of latently-infected T-cells (ACH2) can activate second messenger signaling and increase cell adhesion and migration^46^. Several glycoproteins, e.g. galectin-3-binding protein, L-selectin and neogenin, were significantly elevated in ACH2 secretome^46^. These studies, along with our current findings, underscore the significance of cell-to-cell interactions in regulating latency and/or reactivation of the HIV-1 reservoirs, which may be exploited towards strategies for targeted elimination of these persistent reservoirs. Our findings indicate that MSCs will migrate to and reactivate the latent provirus in U1 cells, and these activities may be further increased following exposure to factors secreted by these latently infected cells.

Although, in our current study, we have not identified the specific MSC-secreted factor(s) that are responsible for latency-reactivation from both U1 & ACH2 cells, future proteomic analysis may facilitate their identification. Due to their therapeutic value, a number of recent studies have conducted proteomic analysis of factors secreted by MSCs, both soluble factors^47^ and those secreted via exosomes^48^. These studies document several cytokines and chemokines which may be involved in activating the PI3K & NFκB pathways in lymphoid cells^49–51^. Our studies using signal transduction inhibitors is of critical importance and may help in the discovery of novel LRAs that may be used alone or in conjunction with currently approved LRAs. Indeed, our findings on the crucial roles of PI3K and NFκB signaling pathways and the negative regulatory effect of the AKT pathway in MSC-mediated latency reactivation, clearly indicate the potential to design superior reservoir targeted agents.

The translational significance of our findings was documented by the coexposure of U1 cells to ASC-conditioned media (ASC-CM) and other LRAs. We observed that ASC-CM significantly increases the latency-reactivation ability of PMA, a PKC-agonist (Figs 1C and 4E) and panobinostat, a HDACi (Fig. 6). Therefore, factors secreted by MSCs, or specific signaling pathways activated by primary human MSCs, may provide novel and superior strategies to activate the latent tissue reservoirs, and will no doubt be of more physiologic significance^33,38,52^. In this respect, several laboratories have shown that the combination of LRAs with different mechanisms of action may be more effective and less toxic *in vivo*^37,38,53^. Coexposure to bryostatin (a PKC-agonist) and panobinostat (a HDACi) provided better latency reactivation, as compared to each of these agents alone^53^.

Although the role of PI3K signaling in provirus reactivation has not been well investigated, a recent study has shown that an activator of PI3K signaling, compound 57704 [1,2,9,10-tetramethoxy-7H-dibenzoquinolin-7-one] can reactivate HIV-1 in several cell-line models of latency, e.g. U1, J89GFP and ACH-2^54^. This quinolinone compound was also found to be a specific agonist of the PI3K p110α isoform, but not the p110β, δ or γ isoforms^54^. Another recent study by Kumar *et al*., (2017) documented that the mammalian target of rapamycin complex 1 (mTORC1), which is a downstream target of PI3K and AKT, is necessary for HIV-1 production from U1 cells^55^. Indeed, similar to our findings using SC-79 (an AKT-activator), which suppressed ASC-mediated latency-reactivation; these investigators demonstrated that inhibition of mTORC1 or PI3K was able to inhibit HIV-1 reactivation as well^55^. Therefore, the crucial role of PI3K pathway, and more specifically, the cross-talk between the PI3K and NFκB pathways, should provide a novel therapeutic approach. Our studies using both PX866 and CDDO-Me showed that ASC-mediated HIV-1 reactivation requires both PI3K and NFκB pathways, and studies using 124005 and SC-79 indicated that latency-reactivation may be negatively regulated by AKT. Interestingly, although the PI3K inhibitor, LY294002 was able to block latency-reactivation, only a slight decrease in total PI3K(p110β) and Nef protein levels were observed, indicating that other PI3K isoforms may be responsible for latency-reactivation via MSCs.

Additionally, our *in vitro* studies showed that the orally available PI3K-inhibitor, PX-866, currently in clinical trials as an anti-cancer agent^56^ and the potent NF-kB inhibitor, CDDO-Me (Bardoxolone-methyl; BM) currently in clinical trials for chronic kidney disease and diabetes^57,58^ may be of significant translational value to target the HIV-1 reservoirs. Our findings on the migration and homing ability of MSCs also implicate the potential of reservoir-tropic MSCs as anti-HIV gene delivery vehicles. Indeed, the tumor-homing ability of MSCs are being actively tested for the delivery of anti-cancer genes to sequestered metastatic sites^59,60^ and thus, the inherent latency-reactivation potential of MSCs and their inherent tropism for viral reservoirs may be exploited towards a novel cell-based gene therapy in HIV-1 infected patients.

## Conclusions

Our findings showed that factors secreted by MSCs, obtained from ten different donors, enabled a rapid and potent HIV-1 reactivation from both latently-infected U1 (monocytic) & ACH2 (T-cells). Furthermore, exposure to factors secreted from these latently-infected cells were able to attract MSCs, and these migratory MSCs (mMSCs) retained their latency-reactivation potential. Coexposure to MSC-secreted factors increased the efficacy of LRAs such as PMA (a PKC agonist) and panobinostat (a HDAC inhibitor). The orally available drugs PX-866 (a PI3K inhibitor) and CDDO-me (a NFκB inhibitor) suppressed MSC-mediated latency-reactivation. Therefore, investigations on the role of tissue-resident MSCs in regulating HIV-1 latency and/or reactivation may provide a physiologically relevant model to understand the reservoir microenvironments *in vivo*. Furthermore, our novel findings on the inductive effects of both PI3K and NFκB pathways and the suppressive effect of AKT pathway in regulating MSC-mediated latency-reactivation may facilitate the discovery of more effective strategies to eliminate the persistent HIV-1 reservoirs in patients.

## Methods

### Cell culture

U1 (Cat#165), ACH2 (Cat#349) and J-Lat(9.2) (Cat#9848) cells were obtained from AIDS Reagent Program, Division of AIDS, NIAID, NIH. U937 cells were purchased from American Type Culture Collection (ATCC, VA, USA). These cell lines were cultured in RPMI-1640 medium supplemented with 10% fetal bovine serum (FBS), 1% penicillin–streptomycin and 2 mM l-glutamine (Sigma-Aldrich; St. Louis, MO). Cells were passaged twice a week at a ratio of 1:5. The human adipose-derived MSCs (ASCs) were purchased from LaCell^TM^, LLC (New Orleans, LA) and the bone-marrow derived MSCs (BMSC) were obtained from Tulane University Stem Cell Core (New Orleans, LA). Both ASCs and BMSCs were grown in stromal cell-culture medium (SCM), consisting of DMEM/F12 (Life Technologies; Grand Island, NY) supplemented with 10% FBS (HyClone; Logan, UT) and antibiotics. Fresh medium was added every 2–3 days until 80–90% confluence. Cells were passaged at a 1:4 dilution using 0.25% trypsin/1 mM EDTA (Life Technologies; Carlsbad, CA). Cells were maintained in a humidified incubator containing 5% CO_2_ and 95% air. Cells were cryopreserved under liquid nitrogen in 10% DMSO containing media.

### Antibodies and Chemicals

The following two antibodies were obtained from AIDS Reagent Program, Division of AIDS, NIAID, NIH: (1) anti-HIV-1 Nef polyclonal from Dr. Ronald Swanstrom (Catalog #2949) and (2) antiserum to HIV-1 Tat from Dr. Bryan Cullen (Cat#705). Antibodies against AKT, phospho-AKT and PI3K p110β were purchased from Cell signaling (Danvers, MA). Antibodies against NFκB p65 & phospho-p65 and GAPDH were purchased from Santa Cruz Biotechnology (Dallas, TX). The NF-κB inducer, phorbol 12-myristate 13-acetate (PMA) was from Abcam (Cambridge, MA) and the NF-κB inhibitor, Bardoxolone-methyl (CDDO-Me) was from Selleckchem (Houston, TX). The AKT inhibitor, 124005, was from Calbiochem (San Diego, CA) and the AKT-inducer, SC79 was from Cayman Chemicals (Ann Arobor, MI). The HDAC inhibitors, vorinostat and panobinostat were also purchased from Cayman Chemicals. The PI3K inhibitors, LY294002 and PX-866 were from Selleckchem and Cayman Chemicals, respectively. Drugs were reconstituted in DMSO, stored in aliquots at −80 °C, and diluted in media immediately before use.

### Adipogenic differentiation

ASCs (1 × 10^5^ cells) were culture in 6-well plates till 80–90% confluent. Cells were grown under either control SCM media or the adipogenic differentiation medium (ADM). The ADM consisted of SCM media supplemented with 0.5 mM isobutylmethylxanthine (IBMX), 50 μM indomethacin and 0.5 μM dexamethasone^61^. Complete media was replenished every three days. After 12 days, cells were fixed with 70% ethanol and stained with 0.5% Oil Red-O to verify adipogenic differentiation^61^. In parallel plates, conditioned media (CM) were collected from either ASCs (ASC-CM) or differentiated adipocytes (AD-CM) and stored at −20 °C for later use. The latency-reactivation ability of both ASC-CM and AD-CM were compared.

### Scratch wound assay

Changes in migratory ability of ASCs were measured using Scratch wound assays^62^. Briefly, ASCs were allowed to generate a confluent monolayer in 12-well petri dishes. Plates were then scored with a sterile p200 pipette tip to enable a wound of approximately 0.4–0.5 mm in width. Culture medium was replaced with either serum free medium (SFM) or with the CM from cells, either U937-CM or U1-CM. ASCs were allowed to migrate at 37 °C for 48 h. Images were acquired using a phase-contrast microscope and wound-widths measured using the Image-J software (version 1.50). Results from quadruplicate wells were analyzed.

### Transwell migration assay

Migration of ASCs towards U1-CM was assessed by using a transwell chambers (TWC) containing polycarbonate membrane inserts (8 μm pore) from Corning (Cambridge, MA). Briefly, ASCs (3.0 × 10^4^ cells/500 μL) were seeded in the upper chamber of TWCs and allowed to migrate toward the lower chamber containing either serum free media (SFM) or CM from either U937 cells or U1 cells (U937-CM or U1-CM, respectively). After 24 h, non-migratory cells were carefully removed from the upper chamber and cells attached to bottom of the inserts were fixed (70% methanol) and stained (0.5% crystal violet in 20% methanol). Images of stained cells were captured using an inverted bright-field microscope (10X). Migrated cells were enumerated and analyzed by ImageJ (version 1.50). In parallel plates, following the transwell migration assays, ASCs were harvested from the top and bottom parts of TWCs. These cells were then propagated and expanded, and CM was collected from both the non-migratory ASCs (nmASC) and migratory ASCs (mASC) subpopulation. Both the migratory ability and the latency-reactivation potential of ASCs (both nmASCs and mASCs) were compared in subsequent studies.

### Transwell coculture assay

Transwell coculture assays were carried out using 24-well plates containing a 0.4 μm PTFE membrane insert (Corning, NY) to avoid direct contact with ASCs and U1 cells. Equal number of ASCs and U1 cells (10,000 cells each) were cultured in the upper and lower chambers, respectively. The ASCs in the upper chamber was cultured in 300 μl of ASC culture media and the U1 cells in the lower chamber was cultured in 700 μl of U1 culture media. At different time post trans-well coculture, we collected 300 μl of media from the lower chamber and centrifuged to obtain cell free conditioned media from the U1 cells. Reactivation of HIV-1 from the latently-infected U1 cells was then assessed by measuring HIV-1 p24 levels by ELISA.

### Direct coculture assay

Coculture of U1 cells with ASCs were carried out at two different U1:ASC cell ratios; either 1:5 or 1:1. Cells were cocultured in 6-well plates in 3 ml growth media. A part of cell free culture supernatant (500 μL/well) after 3, 5 and 7 days of co-culture were collected and stored at −80 °C to measure HIV-1 p24 by ELISA. In each time point, the U1 cell pellets were resuspended with equal amount of above mentioned fresh media and put them back to the corresponding wells and cultured with the indicated time points.

### HIV-1 p24 ELISA

HIV-1 p24 levels in the culture supernatants were determined by using an enzyme-linked immunosorbent assay (ELISA) kit from ABL Inc. (Rockville, MD) with minor modifications^63^. Briefly, ELISA plates were first washed with the wash buffer (4 times) and then treated with the disruption buffer. Serial dilutions of cell-free supernatants from U1 cells were then added to these activated ELISA plates. Each plate also contained HIV-1 p24 protein standards (provided in the kit) and negative control wells. Wells were covered with plate-sealer tape and incubated at 37 °C for 1 h. Wells were then washed (5 times) and conjugate solution was added and incubated at 37 °C for 1 h. Following another round of washing, the peroxidase substrate was added to each well and incubated at 37 °C for 30 min. After the addition of stop solution, the absorbance of each well (at 450 nm wavelength) was quantified by using a BioTek Synergy^HTX^ plate reader (BioTek Instruments; Winooski, Vermont). The HIV-1 p24 levels (pg/mL) were calculated using the standard curves.

### HIV-1 LTR activity assay

Transcriptional activity of the HIV-1 LTR was evaluated by using a reporter cell line, U937-VRX494 (U-494)^64^. Briefly, U937 cells were transduced with a replication-defective lentiviral vector LV-VRX494, which regulates GFP expression under the control of HIV-1 LTR. In each experiment, U-494 cells were cultured under different treatment conditions, either stimulators or inhibitors. Flow cytometric analysis of GFP levels was carried after 48 h by using a BD LSR-II flow cytometer (BD Biosciences; San Jose, CA). Images of cells were also captured by using an inverted bright-field microscope (20X). Both the number of GFP positive cells and their mean fluorescence intensities (MFI) were quantified.

### Western immunoblot

Immunoblot analysis was performed according to our previous published protocols^62^. Briefly, cells were lysed in RIPA buffer from Santa Cruz biosciences (Santa Cruz, CA). Protein contents were quantified by using a BCA protein assay kit from Thermo Scientific (Waltham, MA). Equal amount of protein extracts was electrophoresed in 10% SDS-PAGE gels. Separated proteins were transferred onto a nitrocellulose membrane, treated with a blocking solution for 2 h at room temperature, and then incubated with the primary antibody at 4 °C for overnight. Next day, membranes were washed and incubated with horse radish peroxidase (HRP)-conjugated secondary antibody for 1 h at room temperature. Membranes were then exposed to Supersignal^TM^ west femto substrate (Thermo Scientific) and scanned using ImageQuant LAS-500 (GE Healthcare; Princeton, NJ).

### Statistical analysis

All data were summarized using descriptive statistics such as mean, and standard deviation. The analysis of variance (ANOVA) method was used to compare mean differences. Where meaningful, results were presented graphically. The study hypotheses were tested at 5% level of significance throughout the analysis. Significant changes from control values were determined by using a two-tailed student’s t-test and comparison between groups was carried out by ANOVA.
